# Lipoprotein Profile in Immunological Non-Responders PLHIV after Antiretroviral Therapy Initiation

**DOI:** 10.3390/ijms23158071

**Published:** 2022-07-22

**Authors:** Jenifer Masip, Rosa Jorba, Miguel López-Dupla, Pere Domingo, Yolanda María Pacheco, Graciano García-Pardo, Esteban Martínez, Consuelo Viladés, Sergi Veloso, Verónica Alba, Montserrat Olona, Francesc Vidal, Frederic Gómez-Bertomeu, Joaquim Peraire, Anna Rull

**Affiliations:** 1 Infection and Immunity Research Group (INIM), Universitat Rovira i Virgili, 43003 Tarragona, Spain; jenifer.masip@urv.cat (J.M.); rjorba.hj23.ics@gencat.cat (R.J.); jmlopezdupla.hj23.ics@gencat.cat (M.L.-D.); ggarciap.hj23.ics@gencat.cat (G.G.-P.); cvilades.hj23.ics@gencat.cat (C.V.); sveloso.hj23.ics@gencat.cat (S.V.); veronica.alba@urv.cat (V.A.); molona.hj23.ics@gencat.cat (M.O.); fvidalmarsal.hj23.ics@gencat.cat (F.V.); ffgomez.hj23.ics@gencat.cat (F.G.-B.); 2 Infection and Immunity Research Group (INIM), Institut Investigació Sanitària Pere Virgili (IISPV), 43003 Tarragona, Spain; 3 Infection and Immunity Research Group (INIM), Hospital Universitari de Tarragona Joan XXIII, 43005 Tarragona, Spain; 4 Centro de Investigación Biomédica en Red de Enfermedades Infecciosas (CIBERINFEC), Instituto de Salud Carlos III (ISCIII), 28029 Madrid, Spain; estebanm@clinic.cat; 5 Infectious Diseases Unit, Hospital de la Santa Creu i Sant Pau, 08025 Barcelona, Spain; pdomingo@santpau.cat; 6 Immunology Lab, Institute of Biomedicine of Seville, University Hospital Virgen del Rocío/CSIC/University of Seville, 41013 Seville, Spain; ypacheco-ibis@us.es; 7 Infectious Diseases Service, Hospital Clinic-IDIBAPS, University of Barcelona, 08036 Barcelona, Spain

**Keywords:** antiretroviral therapy, cardiovascular risk, immunological non-responder, lipoproteins, longitudinal analysis, nuclear magnetic resonance, people living with HIV

## Abstract

Nuclear magnetic resonance (NMR)-based advanced lipoprotein tests have demonstrated that LDL and HDL particle numbers (LDL-P and HDL-P) are more powerful cardiovascular (CV) risk biomarkers than conventional cholesterol markers. Of interest, in people living with HIV (PLHIV), predictors of preclinical atherosclerosis and vascular dysfunction may be associated with impaired immune function. We previously stated that immunological non-responders (INR) were at higher CV risk than immunological responders (IR) before starting antiretroviral therapy (ART). Using Liposcale^®^ tests, we characterized the lipoprotein profile from the same cohort of PLHIV at month 12 and month 36 after starting ART, intending to explore what happened with these indicators of CV risk during viral suppression. ART initiation dissipates the differences in lipoprotein-based CV risk markers between INR and IR, and only an increase in the number of HDL-P was found in INR + IR when compared to controls (*p* = 0.047). Interestingly, CD4^+^ T-cell counts negatively correlated with medium HDL-P concentrations at month 12 in all individuals (ρ = −0.335, *p* = 0.003). Longitudinal analyses showed an important increase in LDL-P and HDL-P at month 36 when compared to baseline values in both IR and INR. A proper balance between a proatherogenic and atherogenic environment may be related to the reconstitution of CD4^+^ T-cell count in PLHIV.

## 1. Introduction

The incidence of non-acquired immunodeficiency syndrome (AIDS)-defining diseases (nonADDs), such as cardiovascular events (CVEs), is two-fold higher in persons living with human immunodeficiency virus (PLHIV) than in the general population [1,2,3], and this incidence of disease is explained mainly by HIV-induced activation of inflammatory and coagulation pathways [4]. Moreover, combination antiretroviral therapies, from previous exposure to first-generation nucleoside analogue reverse transcriptase inhibitors (NRTIs) and protease inhibitors (PIs), were associated with an increased risk of cardiovascular disease (CVD) [5]. Treated HIV-infected adults have approximately double the levels of inflammatory markers, such as inflammatory interleukin-6, soluble CD14 and soluble CD163, which have been associated with an increased risk of coronary artery inflammation and atherosclerosis when compared to age- and sex-matched healthy populations [4,6,7]. Furthermore, in PLHIV, markers of preclinical atherosclerosis and vascular dysfunction may be associated with impaired immune function [8]. Indeed, in our previous group study, we stated that ART-naïve PLHIV with less than 200 CD4^+^ T-cell counts/μL, which revealed a poor immune response to ART (immunological non-responders), were at a higher cardiovascular (CV) risk than PLHIV who started ART with less than 200 CD4^+^ T-cell counts/μL, and reached more than 250 CD4^+^ T-cell counts/μL at month 36 on ART (immunological responders) [9].

Abnormal levels of blood lipids, such as high concentrations of low-density lipid cholesterol (LDL-C) and low levels of high-density lipid cholesterol (HDL-C), are traditionally known as important predictors of cardiovascular risk. Of interest, the recent use of nuclear magnetic resonance (NMR)-derived lipoprotein subclasses (VLDL, LDL and HDL) for the characterization of lipoprotein particles, including size and particle number, has improved the risk stratification for subclinical atherosclerosis in comparison to conventional lipid measurements [10,11,12,13]. Concretely, NMR-based advanced lipoprotein tests have demonstrated that LDL and HDL particle numbers (LDL-P and HDL-P) are more powerful cardiovascular risk biomarkers than conventional cholesterol markers [14]. Indeed, LDL-P, and more specifically the increased presence of small LDL-P, was found to be associated with a higher atherosclerotic risk [15], whereas an important representation of HDL-P in circulation seemed to be inversely associated with carotid intima–media thickness [16]. Thus, the recent methodology based on a two-dimensional spectra (2D) from diffusion-ordered NMR spectroscopy (DOSY) experiments (Liposcale^®^ test) provides not only lipid concentration but also lipoprotein size, and lipoprotein particle numbers of the main fractions and subclasses compared to liquid chromatography/tandem mass spectrometry (LC-MS/MS) which also offers a valuable tool for determining a lipid profile with potential diagnostic applications due to its sensitivity and rapid separation limited to lipid concentrations [17,18].

Thus, using a two-dimensional NMR technique (Liposcale^®^) [9], we previously reported higher baseline values of both LDL particles/HDL particles and total particles/HDL particle ratios in INR than in IR [9]. These results support the idea that chronic activation of the innate immune system could be related to increased CVD in PLHIV with poor immune responses. However, information regarding the evolution of lipoprotein lipid content and particle distribution once ART was introduced to INR or, more importantly, how ART could improve or impair the suspected baseline CV risk is still scarce. Thus, in the current study, we aimed to characterize the lipoprotein evolution from INR and IR during ART and to corroborate whether LDL-P/HDL-P or total particles/HDL-P ratios, both indicators of CV risk, remain significantly higher in INR compared to IR during ART follow-up. For that reason, using the same 2D ^1^H-NMR (Liposcale^®^) and the same study cohort, we evaluated the lipoprotein profile at months 12 and 36 [9], and we also included a group of PLHIV starting ART with more than 200 CD4^+^ T-cell counts/μL to explore whether the ART-naïve CD4^+^ T-cell decrease could affect cardiovascular health in PLHIV once ART was introduced.

## 2. Results

**Figure 1:**
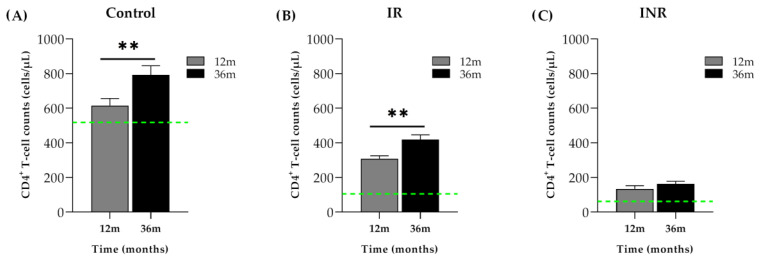
CD4^+^ T-cell counts during ART. CD4^+^ T-cell counts (cells/μL) at month 12 and month 36 in (**A**) control group starting first ART with more than 350 cells/µL, (**B**) immunological responders (IR) who presented with a CD4^+^ T-cell count equal to or greater than 250 cell/µL at month 36 and (**C**) immunological non-responders (INR) who did not reach more than 250 cell/µL CD4^+^ T-cell at month 36. Horizontal green dotted lines represent baseline values of the CD4^+^ T-cell counts in each group. The Wilcoxon paired test was performed to evaluate differences between month 12 and month 36. ** *p* < 0.001.

A total of 21 PLHIV with good recovery status at the initiation of ART (472 (383–563) cells/µL) were included in the study as a control group. Of the 53 PLHIV with low CD4^+^ T-cell counts at the initiation of ART (less than 200 cells/µL), 38 had a successful good recovery status at month 36 (IR). In IR, the total CD4^+^ T-cell counts were four times increased at 36 months (357 (303–461.8) cells/µL) compared to baseline values (90 (45–171) cells/µL) (*p* < 0.001), but the values remained significantly lower than the control group at the same time point (month 36) (752 (639–864) cells/µL (*p* < 0.001)). However, 15 participants of the 53 initiating ART with less than 200 cells/µL did not reach a good recovery status even after being on successful virological suppression (INR). In INR, the mean total CD4^+^ T-cell counts at month 36 were also significantly increased (186 (117–220) cells/µL) when compared to their baseline values (57 (27–99) cells/µL, *p* < 0.001), although the values prevailed significantly lower when compared to the total CD4^+^ T-cell counts of both IR and controls (*p* < 0.001) (Figure 1A).

### 2.1. Stable ART Diminishes Differences in Lipoprotein-Based Cardiovascular Risk Markers

Previously, both total particles/HDL-P and LDL-P/HDL-P ratios confirmed that INR participants were at higher risk of suffering cardiovascular diseases than IR participants before starting ART [9]. Thus, both predictive cardiovascular markers were evaluated again at month 12 and month 36, once ART was initiated, and were compared to the control group. No significant differences were observed among groups at month 12 or at month 36.

**Figure 2:**
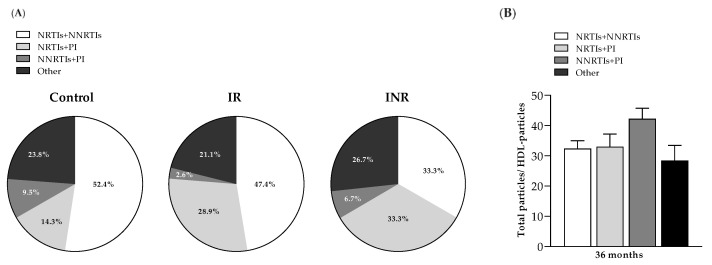
Evaluation of cardiovascular risk by ART scheme. (**A**) Percentage distribution of the different ART schemes including the combinations of nucleoside analogue reverse transcriptase inhibitors (NRTIs) plus a nonnucleoside reverse transcriptase inhibitor (NNRTI) or protease inhibitors (PIs) or the combination of NNRTI +PI in the study group. (**B**) Total particle/HDL particle ratio at month 36 in the recategorized study group (*n* = 74) based on the combination of ART received. To analyze the lipoprotein profile evolution during ART follow-up, data at month 12 and data at month 36 were normalized by dividing each item by its respective baseline value.

Then, we investigated whether the type of ART prescribed differentially affects the CV risk by comparing the ratios in the recategorized study cohort based on the ART received. At month 36, the percentage of antiretroviral therapies based on the combination of nucleoside analogue reverse transcriptase inhibitors (NRTIs) plus nonnucleoside analogue reverse transcriptase inhibitors (NNRTIs) was the primary modality for the treatment and management of HIV infection in all groups, which was followed by ART schemes containing NRTIs plus protease inhibitors (PIs) (Figure 2A). Although both ratios were higher in HIV-positive patients receiving NNRTI + PI, no statistically significant results were obtained when compared to other ART schemes (Figure 2B). Of note, the NNRTI+PI was the less-frequent ART combination in the controls, IR and INR (Figure 2A).

### 2.2. HDL Particle Size Correlates with CD4^+^ T-Cell Count at Month 12 (ART)

**Figure 3:**
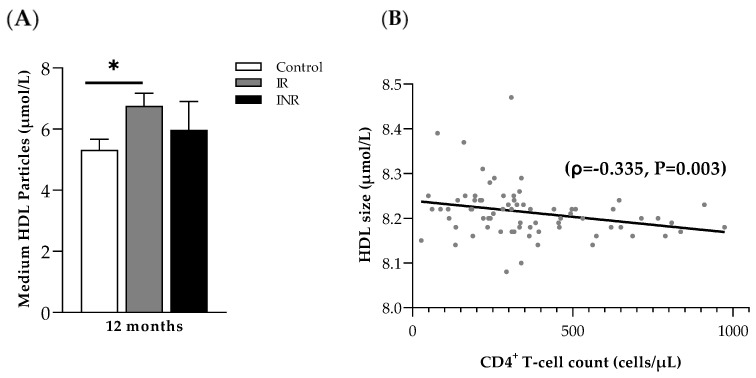
The negative relationship between HDL particle size and CD4^+^ T-cell count. (**A**) HDL medium-size particle distribution (μmol/L) at month 12 in each studied group (control group starting first ART with more than 350 cells/µL, immunological responders (IR) who presented CD4^+^ T-cell count equal to or greater than 250 cells/µL at month 36 and immunological non-responders (INR) who did not reach more than 250 cell/µL CD4^+^ T-cell at month 36). Mann–Whitney nonparametric test * *p* < 0.05. (**B**) Spearman correlation analysis between HDL size (μmol/L) and CD4^+^ T-cell counts (cells/µL) in all individuals (*n* = 74) (ρ = −0.335, *p* = 0.003).

Regarding the lipid concentration and lipoprotein sizing and particle number determination, only differences in the HDL particles were statistically significant at month 12 between controls, IR and INR. Concretely, the size of HDL particles was significantly higher in those individuals starting ART with less than 200 cells/µL than in controls (21.38 (17.87–24.34) µmol/L and 19.56 (14.80–21.86) µmol/L, respectively; *p* = 0.047), probably due to the differences in the number of medium HDL particles (6.15 (4.74–7.80) µmol/L and 5.07 (4.26–6.44) µmol/L, respectively, *p* = 0.06). Indeed, although the number of medium HDL particles was significantly higher in IR and INR at month 12 (6.64 (5.1–7.71) µmol/L) compared to controls, only the differences between IR and controls were statistically significant (*p* = 0.015) (Figure 3A). Correlation analyses confirmed the negative association between CD4^+^ T-cell counts and HDL size at month 12 (ρ = −0.335, *p* = 0.003) (Figure 3B). At month 36, no statistically differences were found in lipid concentration. For both lipoprotein sizing and particle number determination.

### 2.3. Lipoprotein Profile Evolution in PLHIV Based on the 2D ^1^H-NMR Liposcale Test

As mentioned before, the baseline lipoprotein signature based on the two-dimensional spectra from the diffusion-ordered NMR spectroscopy (DOSY) experiment was previously described for INR and IR participants [9]. At baseline, total particles/HDL-P and LDL-P/HDL-P ratios were 1.42-times and 1.39-times higher in INR than in IR, respectively. Thus, to analyze the lipoprotein profile evolution during ART follow-up, the lipoprotein profile obtained during ART was normalized by dividing each item (month 12 and month 36) by its respective baseline values.

#### 2.3.1. Lipoprotein Profile Evolution in PLHIV Starting ART with More than 350 CD4^+^ T-Cells/µL

**Figure 4:**
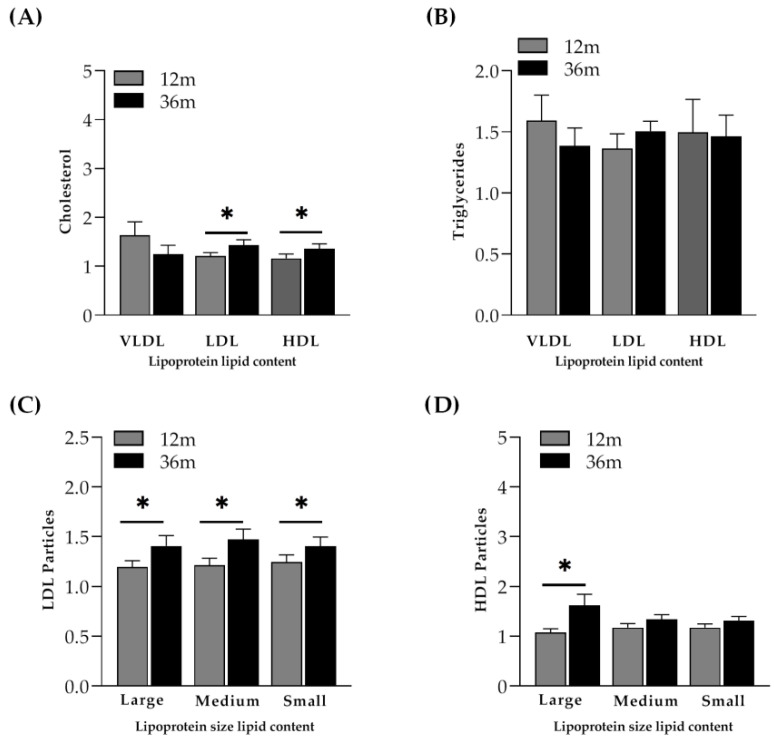
Cholesterol and triglyceride content and lipoprotein particle size distribution. (**A**) Distribution of cholesterol content in VLDL, LDL and HDL at month 12 and month 36 in the control group starting first ART with more than 350 cells/µL. (**B**) Distribution of triglyceride content in VLDL, LDL and HDL at month 12 and month 36. (**C**) Lipoprotein particle size distribution of LDL particles (large, medium and small (nmol/L)) at month 12 and month 36 (**D**) Lipoprotein particle size distribution of HDL particles (large, medium and small (μmol/L)) at month 12 and month 36. Cholesterol content (mg/dL), triglyceride content (mg/dl), LDL particle size (mmol/L) and HDL particle size (µmol/L) at month 12 and month 36 were normalized by dividing each item by its respective baseline value to obtain the ration for each value represented by bars (mean ± SEM). The Wilcoxon paired test was performed to evaluate differences between month 12 and month 36 * *p* < 0.05.

Increases in the mean total cholesterol (1.27 times, *p* < 0.001) and total triglycerides (1.26 times, *p* = 0.013) were found when lipids were assessed with NMR in the control group. Concretely, a higher content of cholesterol in LDL and HDL particles was found to be statistically significant between month 12 and month 36 (*p* = 0.013 and 0.030, respectively) (Figure 4A). The distribution of LDL composition compared to baseline values (LDL-P (*p* = 0.002)) was as follows: the large LDL-P was 1.29 times increased (*p* = 0.003), the medium LDL-P was 1.36 times increased (*p* = 0.002) and the small LDL-P was 1.31 times increased (*p* = 0.001). In fact, all LDL-sized particles showed a significant increase between month 12 and month 36 (Figure 4C). However, the distribution of HDL composition (HDL-P (*p* = 0.003)) compared to baseline values was as follows: the large HDL-P was 1.35 times increased (*p* = 0.004), the medium HDL-P was 1.22 times increased (*p* = 0.003) and the small HDL-P was 1.21 times increased (*p* = 0.011). In this case, only the large-sized HDL particles were found to be significantly increased between month 12 and month 36 (*p* = 0.021) (Figure 4D).

#### 2.3.2. Lipoprotein Profile Evolution in PLHIV Starting ART with Less than 200 CD4^+^ T-Cells/µL

##### Immunological Responders (IR)

**Figure 5:**
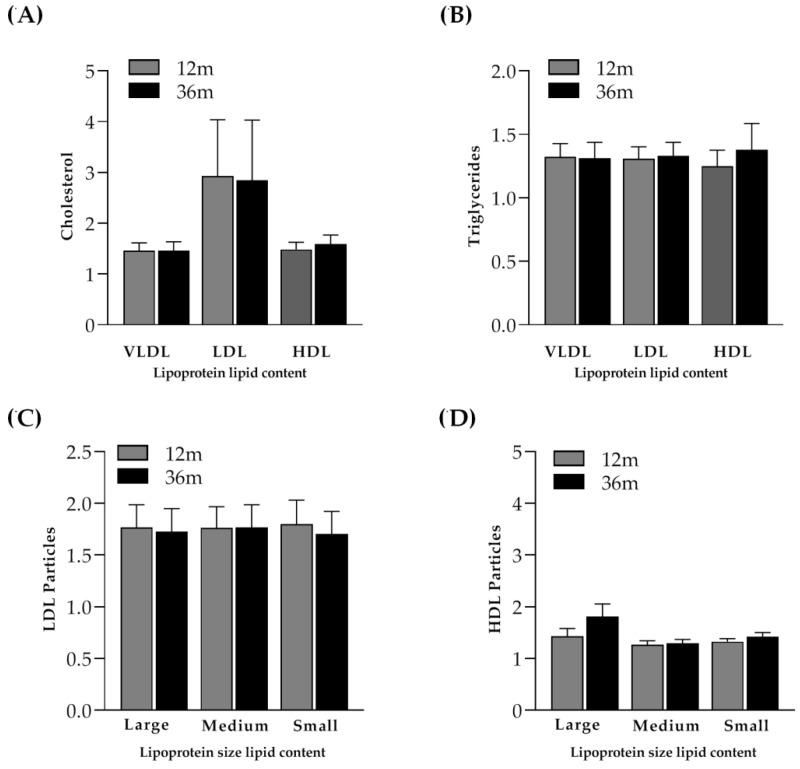
Cholesterol and triglyceride content and lipoprotein particle size distribution. (**A**) Distribution of cholesterol content in VLDL, LDL and HDL at month 12 and month 36 in immunological responders (IRs) who presented with a CD4^+^ T-cell count equal to or greater than 250 cell/µL at month 36. (**B**) Distribution of triglyceride content in VLDL, LDL and HDL at month 12 and month 36. (**C**) Lipoprotein particle size distribution of LDL particles (large, medium and small (nmol/L)) at month 12 and month 36. (**D**) Lipoprotein particle size distribution of HDL particles (large, medium and small (μmol/L)) at month 12 and month 36. Cholesterol content (mg/dL), triglyceride content (mg/dL), LDL particle size (mmol/L) and HDL particle size (µmol/L) at month 12 and month 36 were normalized by dividing each item by its respective baseline value and ratios are represented by bars (mean ± SEM). The Wilcoxon paired test was performed to evaluate differences between month 12 and month 36.

IR participants showed a higher mean total cholesterol at month 36 compared to baseline values due to a significant increase in the content of cholesterol in all lipoproteins (1.25 times in VLDL (*p* = 0.014), which was 1.40 times in LDL (*p* < 0.001) and 1.36 times in HDL (*p* < 0.001)). However, no significant differences were found in the cholesterol and triglyceride content between month 12 and month 36 (Figure 5A,B). Similar to the control group, the distribution of large HDL particles increased from month 12 to month 36, although the differences were not statistically significant (Figure 5D).

##### Immunological Non-Responders (INR)

**Figure 6:**
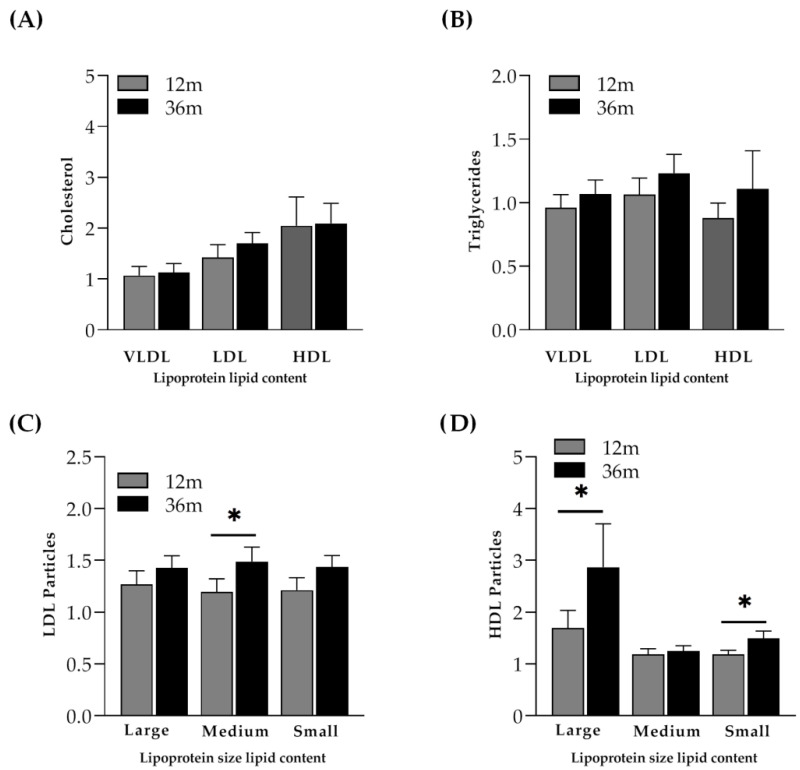
Cholesterol and triglyceride content and lipoprotein particle size distribution. (**A**) Distribution of cholesterol content in VLDL, LDL and HDL at month 12 and month 36 in immunological non-responders (INR) who did not reach more than 250 cell/µL CD4^+^ T-cell at month 36. (**B**) Distribution of triglyceride content in VLDL, LDL and HDL at month 12 and month 36. (**C**) Lipoprotein particle size distribution of LDL particles (large, medium and small (nmol/L)) at month 12 and month 36. (**D**) Lipoprotein particle size distribution of HDL particles (large, medium and small (μmol/L)) at month 12 and month 36. Cholesterol content (mg/dL), triglyceride content (mg/dL), LDL-particle size (mmol/L) and HDL-particle size (µmol/L) at month 12 and month 36 were normalized by dividing each item by its respective baseline value and ratios are represented by bars (mean ± SEM). The Wilcoxon paired test was performed to evaluate differences between month 12 and month 36 * *p* < 0.05.

Similar to controls and IR participants, in INR, the mean LDL cholesterol (*p* = 0.022) and HDL cholesterol (*p* = 0.001) were higher at month 36 when compared to baseline values (both were 1.53 times increased). Interestingly, in that case, a significant increase in the distribution of some LDL particles and some HDL particles was observed. As previously observed in controls, medium LDL particles (*p* = 0.047) and large HDL particles (*p* = 0.041) were significantly higher at month 36 than at month 12 (Figure 6C,D). Of interest, small HDL particles (*p* = 0.047) were significantly higher at month 36 than at month 12 (Figure 6D).

## 3. Discussion

Impaired immune function was associated with markers of preclinical atherosclerosis and vascular dysfunction in PLHIV [8]. Notably, advanced HIV infection (low CD4^+^ counts) linked to chronic inflammation and increased immune activation was associated with the alteration of metabolic parameters related to lipid metabolism and increased atherogenic risk [8,19]. Indeed, in naïve PLHIV, we previously reported an increased CV risk in INR compared to IR, using the lipoprotein signature measured by a 2D ^1^H-NMR (Liposcale^®^) [9]. Because ART was associated with an increased risk of coronary artery inflammation and atherosclerosis compared to the healthy population [4,6,7], we considered it critical to reevaluate the CVD risk from our previously studied cohort of INR and IR after starting ART. Thus, the new analyses were performed with a total of 53 PLHIV from the previous cohort of 64 individuals [9] who were successfully followed up with ART until month 36 and had an available sample for the 2D ^1^H-NMR technique. Both INR and IR were compared to a group of PLHIV who initiated ART with optimal values of CD4^+^ T-cell count and maintained the same period of stable ART at follow-up. Our findings corroborated that the initiation of ART progressively increased LDL-P and LDL-C concentrations in HIV-positive patients. However, both large-sized HDL-P and small-sized HDL-P were noticeably increased from month 12 to month 36 in INR. Remarkably, at month 12, medium HDL particle concentrations were significantly higher in PLHIV starting ART with low CD4^+^ T-cell counts. The negative correlation between CD4^+^ T-cell count and the medium HDL-P could indicate the importance of HDL-P in poor immune recovery status, in agreement with previously published data [20].

Both total particles/HDL particles and LDL particles/HDL particles ratios provide additional predictive information over other used cardiovascular biomarkers [19,21]. Notably, at month 12 and month 36, no significant differences were observed among the groups. The greater predictive value observed in naïve INRs was dissipated with the initiation of ART, suggesting that the introduction of ART could hit PLHIV alike with no dependence on immune status. Moreover, from the recommended ART combinations, the use of NNRTI + PI seemed to have a more negative impact on CVD, although the results were not statistically significant, probably due to the low number of individuals affected by this ART combination.

Both LDL-P concentrations and LDL-C were increased between month 12 and month 36 in controls and INRs, although the differences were only significant in the control group. Of note, medium-sized LDL-P was significantly higher in controls and INRs, whereas large-sized LDL-P and small-sized LDPL-P were significantly higher only in the control group. Although IRs showed no significant differences between month 12 and month 36, high concentrations of both LDL-P and LDL-C were observed during all ART follow-ups. Higher concentrations of LDL-P and LDL-C are considered to be atherogenic. Previous prospective studies showed that the LDL-P number had a better discriminatory role in cardiometabolic risk than LDL-C concentrations since LDL-C could be a consequence of the initiation of the antiretroviral regimen [22]. However, a decrease in large-sized LDL-P followed by an increase in medium- or small-sized LDL-P was previously related to a two-fold and 40% increase in risk for coronary heart disease, respectively [23]. Indeed, small, dense LDL particles have been proven to confer greater atherogenic risk than larger, less dense LDL particles [15].

Large HDL-P concentrations increased from month 12 to month 36 in all HIV-positive patients, whereas in the INR, there was not only a progressive increase in large HDL-P concentrations but also in small HDL-P concentrations. Plasma concentrations of specific HDL subpopulations could be strongly associated with cardiovascular risk when compared to total HDL particle number [24]. Several recent studies have shown the atheroprotective properties of large HDL particles when compared to small and medium HDL particles [25], which typically reveal positive correlations with risk.

Missing information regarding inflammatory-related parameters, immunological recovery factors (CD4/CD8 ratio) and important cardiovascular clinical parameters were the major limitations of the present study. However, connecting information on lipidomics from previous omics studies was also challenging because of the interassay bias that was minimized by the normalization of the data. Moreover, it could also be interesting to continue the follow-up for a longer period for a better conclusion. Validation studies are needed.

## 4. Materials and Methods

### 4.1. Study Design and Participants

**Figure 7:**
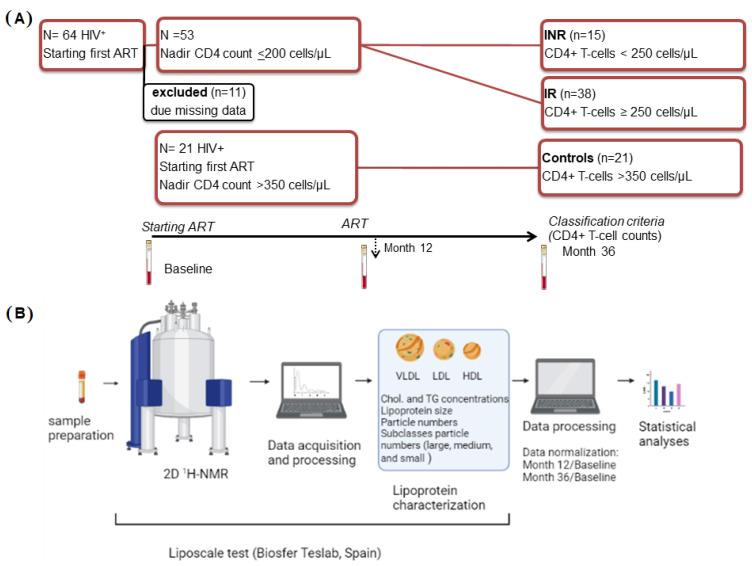
Flowchart illustrating the study design and analysis. (**A**) From the initial cohort of 64 PLHIV starting ART with less than 200 cells/µL, 53 had available follow-up datasets at both month 12 and month 36 on ART. The group of 53 PLHIV were categorized based on their CD4^+^ T-cell count after 36 months of being on stable ART: immunological non-responders (INR) when their CD4^+^ T-cell count was less than 250 cells/µL and immunological responders (IR) when their CD4^+^ T-cell count was equal to or greater than 250 cells/µL. A group of 21 PLHIV starting ART with more than 350 cells/µL was added as a control group. Blood samples were obtained at baseline, and at month 12 and month 36 on ART. (**B**) Using Liposcale^®^ tests (2D 1H-NMR), the lipoprotein profile (including cholesterol (chol.) and triglycerides (TG) concentrations, sizes, particle numbers for each lipoprotein and particle numbers of nine subclasses, namely large, medium, and small VLDL, LDL, and HDL, respectively) was obtained at month 12 and month 36 after starting ART. Before statistical analyses, data at month 12 and data at month 36 were normalized by dividing each item by its respective baseline value. Workflow created with BioRender.com.

This was a multicenter, prospective cohort study comprising all adult HIV-1-infected individuals who started first ART between 2009 and 2011 and were followed up at the HIV outpatient clinics of the participating hospitals: Hospital Joan XXIII (Tarragona), Hospital de la Santa Creu i Sant Pau (Barcelona) and Hospital Virgen del Rocío (Sevilla). Of the initial cohort of 64 participants [9], only 53 HIV patients had available follow-up datasets at both month 12 and month 36 on ART (Figure 7). According to the previous immune recovery classification, the two groups were categorized based on their CD4^+^ T-cell count after 36 months of being on stable ART: the immunological responder group when their CD4^+^ T-cell count was equal to or greater than 250 cells/µL (IR) and immunological non-responders (INR), if they did not reach this threshold. A group of 21 HIV individuals starting with first ART with more than 350 cells/µL and then maintaining the same cutoff of CD4^+^ T-cell count at month 36 was added as a control group.

All the selected patients were required to fulfil the following inclusion criteria: aged ≥ 18 years, presence of HIV-1 infection, on an ART regimen during the 36 months of the study and undetectable plasma HIV-1 viral load at 36 months. The exclusion criteria that could potentially bias the results of the study were the presence of active opportunistic infections, current inflammatory diseases or conditions that could develop a CVD, changes in the ART regimen and the use of drugs known to affect/modify CD4^+^ T-cell counts (antineoplastic drugs, steroids, immune response modulators, and colony-stimulating factors, among others).

### 4.2. Laboratory Measurements

Blood was drawn from a peripheral vein after an overnight fast, collected in ethylenediaminetetraacetic acid (EDTA) tubes and immediately centrifuged for 15 min at 4 °C and 1500× *g*. Plasma samples were then stored at −80 °C at the BioBanc IISPV until further analysis.

### 4.3. D 1H-NMR Liposcale Test

The 2D ^1^H-NMR Liposcale test (Biosfer Teslab, Reus, Spain) was previously used to characterize INR and IR participants before starting ART [9]. The Liposcale test provides lipid concentrations (triglycerides and cholesterol), sizes, and particle numbers for VLDL, LDL, and HDL classes, as well as the particle numbers of nine subclasses, namely large, medium, and small VLDL, LDL, and HDL, respectively. The test was based on two-dimensional spectra (2D) from diffusion-ordered NMR spectroscopy (DOSY) experiments to decompose the (CH_3_) proton resonances of the lipoprotein particles (BrukerAvance III 600 spectrometer (Bruker GmbH, Karlsruhe, Germany), operating at a proton frequency of 600.20 MHz (14.1 T), at 310 K) into nine Lorentzian functions [12]. The methyl signal was surface-fitted and the number of functions was increased to account for the nine lipoprotein subclasses. Cholesterol and triglyceride concentrations of the main lipoprotein fractions (VLDL, LDL and HDL) were predicted using partial least squares (PLS) regression models. The NMR functions were associated with a given lipoprotein class according to their associated NMR size. The main lipoproteins fractions were defined as VLDL (38.6–81.9 nm), LDL (18.9–26.5 nm) and HDL (7.8–11.5 nm). The mean particle size of every main fraction (VLDL, LDL and HDL) was derived by averaging the NMR area of each fraction by its associated size. Particle-weighted lipoprotein size was obtained by dividing each NMR area by its associated volume. For each lipoprotein class, the mean particle size was obtained by multiplying the NMR lipoprotein particle size by its fractional particle concentration relative to the total particle concentration of a given class [12]. The particle numbers of each lipoprotein main fraction were calculated by dividing the lipid volume by the particle volume of a given class. The lipid volumes were determined by using common conversion factors to convert concentration units obtained from the PLS model into volume units. The relative areas of the lipoprotein components used to decompose the 2D spectra were used to derive the particle numbers of the nine lipoprotein subclasses.

### 4.4. Statistical Analysis

All data were tested for normality using the Kolmogorov–Smirnov test before the statistical analyses were applied. A descriptive data analysis of patient characteristics was carried out using percentages for categorical variables and medians and interquartile ranges (25th percentiles–75th percentiles) for continuous variables. The “times increased” data were calculated as a subtraction between the results at month 36 and the respective baseline values. Differences between the groups of the study were assessed through the nonparametric Kruskal–Wallis (KW) and/or Mann–Whitney test for continuous unpaired variables. CD4^+^ T-cell intragroup results among the different time points studied (baseline, month 12 and month 36) were compared using the Friedman and Wilcoxon test for paired samples. To analyze the lipoprotein profile evolution during ART follow-up, the Liposcale test results at month 12 and month 36 were normalized by dividing each item by its respective baseline value. Correlation analysis between CD4^+^ T-cell counts (cells/µL) and lipoprotein items was performed using the Spearman correlation test. The statistical software used was SPSS Software v22 (IBM, Madrid, Spain). Graphs were generated using GraphPad Prism (GraphPad Prism version 5.0 for Windows, GraphPad Software, San Diego, CA, USA). *p* values less than 0.05 were considered statistically significant.

## 5. Conclusions

The present results corroborated the applicability of nuclear magnetic resonance (NMR)-based advanced lipoprotein tests in a population with chronic infection. The initiation of ART progressively increased HDL-P and LDL-P in HIV-positive patients whereas VLDL particles were only increased in IR compared to its baseline values. In INR, ART initiation dissipates the greater CVD risk previously observed at baseline (ART-naïve) when compared with IR. Concretely, in INR, ART follow-up increases large HDL but also large and small LDL-Ps, which denoted a conflict between atherogenic and proatherogenic factors. Additionally, there is a negative correlation between CD4^+^ T-cell counts and medium HDL-P concentrations, corroborating its immunomodulatory role but also its controversial function in CVD. A proper balance between a proatherogenic and atherogenic environment is related to the reconstitution of CD4^+^ T-cell count. Further investigations are needed to elucidate the mechanisms by which the different lipoproteins could be associated to immune response in PLHIV.

## Data Availability

The data that support the finding of this study are available from the corresponding author upon reasonable request.
